# Persistent post-galactogram intraductal iodinated contrast detected on contrast-enhanced mammography (CEM) in a patient with nipple discharge

**DOI:** 10.1016/j.radcr.2022.01.040

**Published:** 2022-02-03

**Authors:** Shao Zun Chen, Timothy B. Rooney, Matthew M. Miller

**Affiliations:** University of Virginia Medical Center, 1215 Lee Street, Charlottesville, VA 22903, USA

**Keywords:** Galactogram, Contrast-enhanced mammography, Nipple discharge

## Abstract

Nipple discharge is a common complaint among adult women and is often evaluated by galactography. Contrast-enhanced mammography (CEM) is an emerging breast imaging modality that is useful in the evaluation of patients with nipple discharge who have a negative galactogram, especially if they are not good candidates for contrast-enhanced MRI. Here we present a case of a 37-year-old female who was 22 weeks pregnant and presented with suspicious nipple discharge. The patient initially underwent galactography, which was negative, and was subsequently referred for CEM for further evaluation. One week after the galactogram, the patient underwent CEM which revealed persistent intraductal iodinated contrast from the galactogram. The retained intraductal contrast obscured the area of concern on the CEM and limited evaluation for underlying areas of enhancement. Given the increasing popularity of CEM in breast imaging practice and its utility in the evaluation of patients with nipple discharge, recognition of retained intraductal contrast as a source of artifact on CEM is important so that steps can be taken to prevent acquiring a limited and/or non–diagnostic CEM. We suggest several practical steps the radiologist can take when planning the diagnostic workup of patients with nipple discharge to ensure the patient will be able to successfully undergo CEM, if needed. These steps will help reduce unnecessary patient exposure to radiation and intravenous contrast and avoid a delay in diagnosis and treatment.

## Introduction

Nipple discharge is a common complaint among adult women, and it can be benign or associated with underlying malignancy [1]. Galactography (or ductography) can be a useful technique for evaluating intraductal lesions in patients with negative diagnostic mammography and ultrasound. In galactography, iodinated contrast material is introduced into the ductal system through cannulation prior to mammographic imaging. Findings suspicious for underlying malignancy include ductal filling defects, obstruction, and wall irregularities. The sensitivity and specificity of galactography, however, is limited due to overlapping structures on conventional mammograms and its inability to reliably distinguish benign and malignant lesions [2].

Patients with nipple discharge can also be evaluated with contrast-enhanced MRI or contrast-enhanced mammography (CEM). Contrast-enhanced MRI has been shown to be more sensitive and more specific than galactography and has the benefit of not requiring the presence of nipple discharge at the time of imaging [3,4]. The main limitations of MRI include higher exam cost, lower accessibility, and contraindications to gadolinium contrast. CEM is an emerging breast imaging modality that is gaining popularity given its high sensitivity and specificity, convenience, and often lower cost than MRI [5,6]. CEM is particularly useful for patients who are not good candidates for contrast-enhanced MRI due to pregnancy, gadolinium contrast allergy, claustrophobia, or other factors [7]. CEM utilizes a dual-energy acquisition technique in which both a low-energy image and a high-energy image are obtained following intravenous (IV) administration of iodinated contrast. The low-energy image is similar to a conventional mammogram and demonstrates breast anatomy, while the high-energy image highlights areas of high iodine contrast uptake, which can indicate an underlying abnormality.

Here we present a case of persistent intraductal iodinated contrast visualized 1 week after galactography on a CEM exam performed on a patient with nipple discharge. Significantly, the persistent intraductal contrast limited evaluation for areas of abnormal enhancement on the CEM.

## Case description

A 37-year-old female, 22 weeks pregnant and without personal or family history of breast cancer, presented with a chief complaint of nipple discharge for 2 months. The patient reported spontaneous bloody discharge from the left nipple with no associated breast pain, palpable lump, or skin changes.

**Fig. 1:**
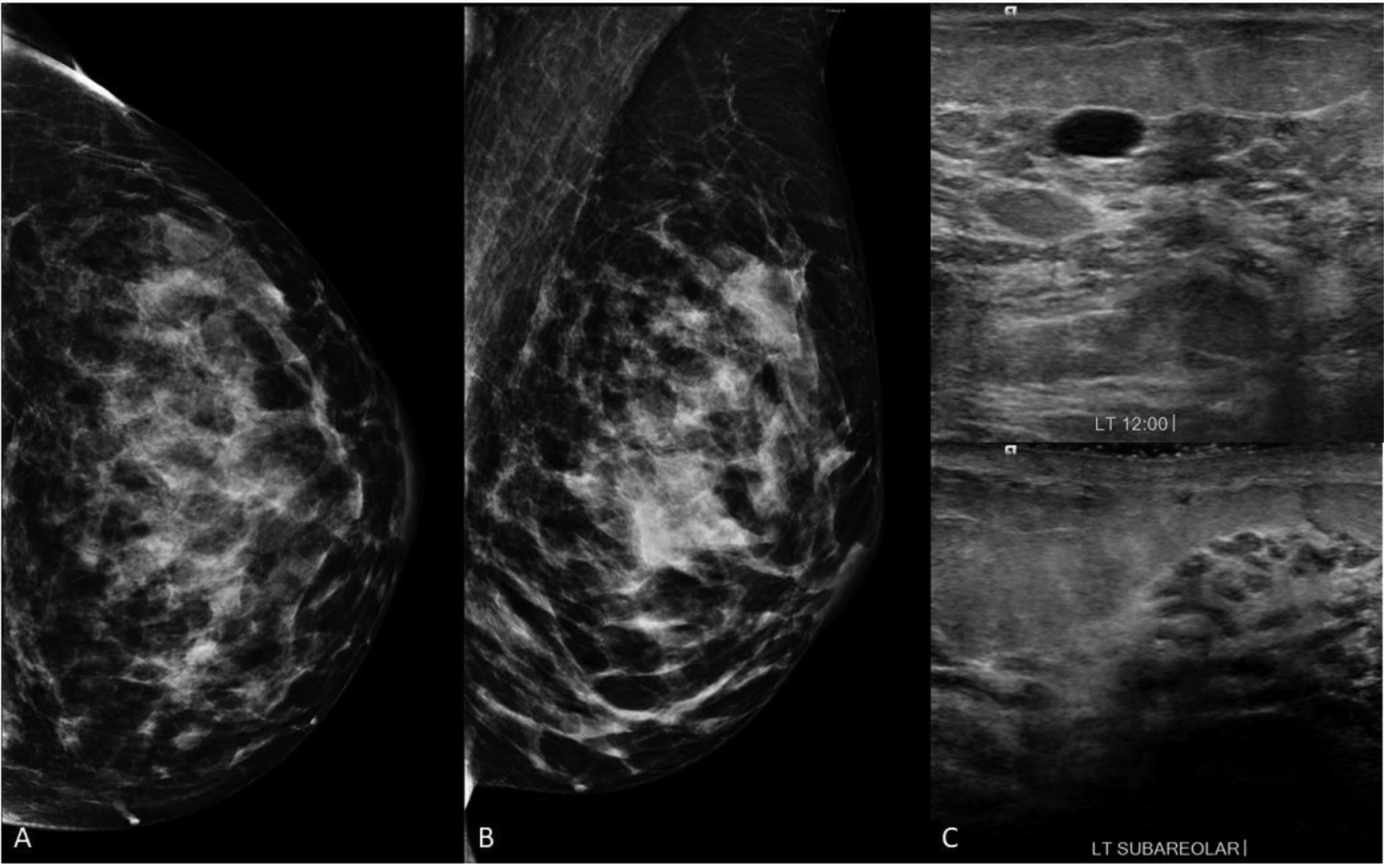
Initial diagnostic imaging in a 37-year-old female with spontaneous bloody left nipple discharge. (A) Craniocaudal (CC) and (B) mediolateral oblique (MLO) views demonstrate multiple bilateral oval circumscribed masses in both breasts (right breast not shown). No abnormality is seen in the subareolar region. (C) Representative image from the diagnostic ultrasound examination demonstrates one of the numerous cysts present in the left breast. No sonographic abnormality was seen in the subareolar left breast.

The initial diagnostic mammogram demonstrated multiple bilateral oval circumscribed masses. The diagnostic ultrasound exam revealed numerous cysts and clustered microcysts with no suspicious mass identified (Fig. 1). The subareolar regions were unremarkable. On physical examination, clear discharge was successfully expressed from a single duct in the left nipple. Galactography was recommended for further evaluation given the concerning history of nipple discharge.

**Fig. 2:**
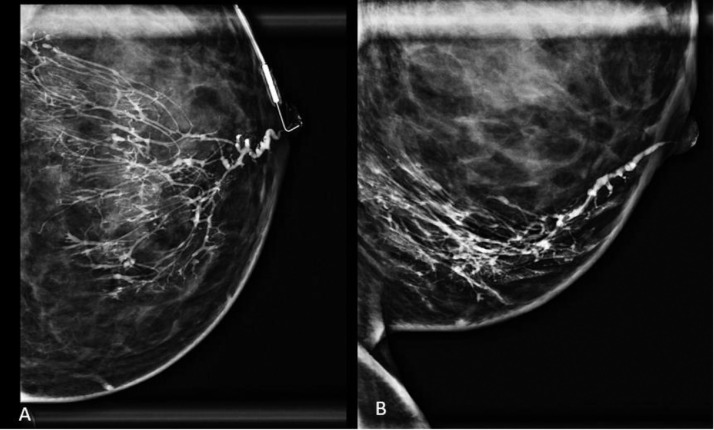
Galactography in a 37-year-old female with spontaneous bloody left nipple discharge. (A) Craniocaudal (CC) and (B) mediolateral (ML) views following single duct cannulation with injection of approximately 0.9 mL of iodinated contrast material demonstrate opacification of a normal ductal system without filling defects, obstructions, or wall irregularities.

The patient returned a week later for galactography, where the single duct from the left central nipple was cannulated using a 30-gauge galactography catheter (Ranfac, Avon, MA), and approximately 0.9 mL of Omnipaque 350 contrast material (GE Healthcare, Marlborough, MA) was injected into the duct. The subsequent galactogram demonstrated a normal appearing duct without filling defects or concerning abnormalities (Fig. 2). Further evaluation with CEM was recommended due to persistent symptoms and the patient's contraindication for contrast-enhanced MRI given her pregnant status.

**Fig. 3:**
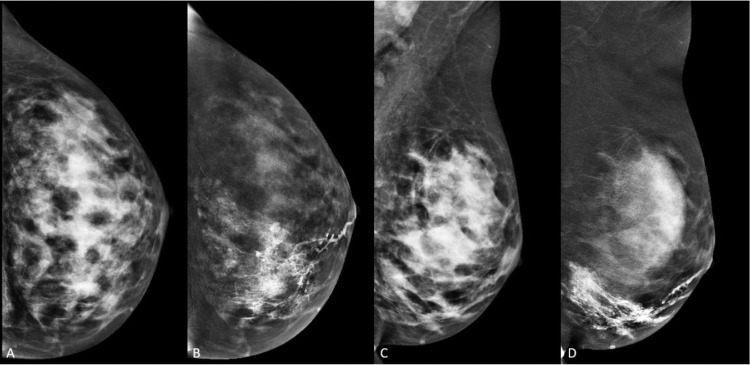
Contrast enhanced mammogram (CEM) in a 37-year-old female with spontaneous bloody left nipple discharge, performed 1 week following galactography. (A, C) Low-energy mammogram images demonstrate no suspicious lesions. (B, D) Recombined high-energy mammogram images (iodine images) demonstrate marked background parenchymal enhancement with persistent intraductal iodinated contrast within a ductal system in the inferior left breast from prior galactography exam. No definite abnormal enhancement is seen within this limitation.

The patient returned 1 week following the galactogram and CEM was performed after IV administration of Omnipaque 350. No radiographic abnormality or enhancing mass was identified in the left breast on CEM, but interpretation of the CEM was significantly limited due to an artifact caused by retained contrast material from the prior galactogram within the ductal system in the inferior left breast (Fig. 3). Interestingly, this intraductal contrast was no longer visible on the conventional mammography image, but created a significant artifact on the iodine image.

Subsequent to the CEM imaging, the patient reported resolution of her suspicious symptoms. She reported that the nipple discharge had ceased to be bloody and had become bilateral and yellow, similar to colostrum. Given that the concerning bloody discharge had resolved and the remaining discharge was likely attributable to the patient's pregnant status, the patient was advised to continue to monitor for symptoms and to follow up clinically with the referring provider. The patient later underwent an uncomplicated delivery and plans to return for follow-up imaging after completion of breastfeeding.

## Discussion

Many radiologic examinations involve the introduction of contrast materials into ductal systems or other body spaces, including examinations of the urinary system (urography and cystography), biliary system (cholangiography), and reproductive system (hysterosalpingography). Galactography is unique among radiologic examinations in that there is no active secretion and/or excretion mechanism that would be expected to clear the contrast material from the intraductal space unless the patient is having active nipple discharge or is breastfeeding. When intraductal contrast from a galactogram extravasates into the adjacent breast parenchyma during injection, a known complication of this procedure, the extravasated contrast material is readily reabsorbed through the rich vascular and lymphatic system in the breast and is usually cleared within 7-14 days [8]. However, in the absence of extravasation, there is no well-documented timing for clearance of contrast material from the ductal system. There are also no documented studies on the effect of retained ductal contrast material in the breast, which may be due to a general lack of awareness of this phenomenon since, as our case illustrates, retained iodinated contrast may be invisible on conventional mammography even when it is readily apparent on the high-energy CEM image.

In our case, the contrast material remained visible within the ductal system 1 week after galactogram on the CEM exam, obscuring the area of concern, and limiting evaluation for underlying areas of enhancement. Given the increasing popularity of CEM in breast imaging practice and its utility in the evaluation of patients with nipple discharge, recognition of retained intraductal contrast material as a source of artifact on CEM is important so that steps can be taken to prevent acquiring a limited, and/or non–diagnostic CEM.

If a patient is likely to undergo CEM rather than MRI for additional diagnostic evaluation in the event of a negative galactogram, several steps could be taken to help ensure that the patient will be able to undergo CEM, if needed. As a first step, it may be beneficial in these cases to minimize the amount of contrast material injected into the duct during the galactogram so that it can be cleared by the body more quickly, if this can be done without compromising the diagnostic utility of the galactogram. As a second step, sufficient time should be allowed to pass after the galactogram before attempting the CEM to allow for adequate ductal clearance. The amount of time to wait would depend on clinical experience and may vary based on the amount of the discharge being produced. A potential third step could be to acquire a single-view “scout” CEM prior to IV contrast administration to evaluate for any residual intraductal contrast that could compromise the diagnostic utility of the CEM. Based on this scout image, the CEM exam could proceed as planned or be rescheduled to another day when the intraductal contrast has cleared.

## Conclusion

Galactography and CEM are both useful tools in the diagnostic workup of patients with nipple discharge. Awareness of the artifact produced on CEM by retained intraductal contrast material from galactography will help the radiologist plan ahead to ensure that the patient will be able to undergo a successful CEM after the galactogram, if needed. This will ultimately allow for prompt diagnosis and appropriate treatment for these patients, while avoiding unnecessary IV contrast administration, and radiation exposure.

## Patient consent

De-identified mammography and galactography images are submitted for this case report. Only patient's age and gender are included in this case report. Formal consent form available upon request.
